# Change of migration patterns and policies across Europe: impact on help-seeking behaviour of migrants and refugees and on their experiences of mental health care in host countries

**DOI:** 10.1192/j.eurpsy.2026.10366

**Published:** 2026-07-31

**Authors:** M. Sundvall

**Affiliations:** The National Centre for Suicide Research and Prevention, Karolinska Institute, Stockholm, Sweden

## Abstract

**Abstract:**

Wars, social upheavals and climate changes cause refugee wawes and shape migration flows affecting the situation of refugees and other migrants in many countries. Changes in migration policies in the host countries have been introduced and wider changes are announced for the coming years. It is vital for researchers and clinicians to reflect on the possible impact of this development on migrants’ health, help-seeking behaviour and the encounter with healthcare.

The author will present material from literature reviews and official statistics. There will be an overview of current research on a number of themes:

- Risk factors related to migration policies for mental ill-health, suicidal behaviour, and suicide in migrants;
- cultural and social factors affecting help-seeking behaviour of migrants;
- experiences of access to mental healthcare as well as the care encounter, including legal restrictions as well as attitudes of and the impact on clinicians.

The varying conditions of migrants in different European countries, especially regarding access to care and reports of discrimination will be sampled from official statistics.

The aim of the presentation is to stimulate and facilitate the formulation of research questions and the planning of continued documentation and continued studies over time. Further, the aim is to enable clinicians to have a deeper understanding of suicidality in their migrant patients, including the impact of social conditions not always known to healthcare staff.

**Disclosure of Interest:**

None Declared

